# Ectopic endometrium in human foetuses is a common event and sustains the theory of müllerianosis in the pathogenesis of endometriosis, a disease that predisposes to cancer

**DOI:** 10.1186/1756-9966-28-49

**Published:** 2009-04-09

**Authors:** Pietro G Signorile, Feliciano Baldi, Rossana Bussani, Mariarosaria D'Armiento, Maria De Falco, Alfonso Baldi

**Affiliations:** 1 Fondazione Italiana Endometriosi, Rome, Italy; 2 Dept Biochemistry, Sect Pathology, Second University of Naples, Naples, Italy; 3 Dept. of Pathology, University of Trieste, Trieste, Italy; 4 Dept. Scienze Biomorfologiche, University of Naples "Federico II", Naples, Italy; 5 Dept Evolutive and Comparative Biology, University of Naples "Federico II", Naples, Italy

## Abstract

**Background:**

Endometriosis is a gynecological disease defined by the histological presence of endometrial glands and stroma outside the uterine cavity. Women with endometriosis have an increased risk of different types of malignancies, especially ovarian cancer and non-Hodgkin's lymphoma. Though there are several theories, researchers remain unsure as to the definitive cause of endometriosis. Our objective was to test the validity of the theory of müllerianosis for endometriosis, that is the misplacing of primitive endometrial tissue along the migratory pathway of foetal organogenesis

**Methods:**

We have collected at autopsy 36 human female foetuses at different gestational age. We have performed a morphological and immunohistochemical study (expression of oestrogen receptor and CA125) on the pelvic organs of the 36 foetuses included en-block and totally analyzed.

**Results:**

In 4 out of 36 foetuses we found presence of misplaced endometrium in five different ectopic sites: in the recto-vaginal septum, in the proximity of the Douglas pouch, in the mesenchimal tissue close to the posterior wall of the uterus, in the rectal tube at the level of muscularis propria, and in the wall of the uterus. All these sites are common location of endometriosis in women.

**Conclusion:**

We propose that a cause of endometriosis is the dislocation of primitive endometrial tissue outside the uterine cavity during organogenesis.

## Background

Endometriosis is a gynecological disease defined by the histological presence of endometrial glands and stroma outside the uterine cavity, most commonly implanted over visceral and peritoneal surfaces within the female pelvis [1,2]. The prevalence of endometriosis in the general female population is 6–10%; in women with pain, infertility or both, the frequency increases to 35–60% [3]. Deep infiltrating endometriosis is a particular form of endometriosis associated with pelvic pain symptoms, located under the peritoneal surface [4,5]. Though there are several theories, researchers remain unsure as to the definitive cause of endometriosis. The most commonly accepted mechanism for the development of peritoneal endometriotic lesions is the Sampson's theory claiming the adhesion and growth of endometrial fragments deposited into the peritoneal cavity via retrograde menstruation [4]. On the other hand, the coelomic metaplasia theory claims that formation of deep endometriosis is caused by metaplasia of the original coelomic membrane, perhaps induced by environmental factors [6-8]. A different theory postulates that endometriosis is caused by little defects of embryogenesis [9,10]. Indeed, during the embryonic stage, the primitive cells migrate and undergo differentiation to form the pelvic organs. In particular, the Müllerian ducts give rise to the female reproductive tract, including the Fallopian tubes, uterus, cervix, and anterior vagina. This organogenesis is controlled and directed by a sophisticated, but still incompletely understood, fetal system including the regulation of the anti-Müllerian hormone signalling pathway [11]. It has been speculated that aberrant differentiation or migration of the Müllerian ducts could cause spreading of cells or tracts of cells in the migratory pathway of foetal organogenesis across the posterior pelvic floor and this could conveniently explain the observation that endometriosis is most commonly and predictably found in the cul-de-sac, utero-sacral ligaments, and medial broad ligaments, although location anywhere might be possible [12]. This theory of developmentally misplaced endometrial tissue is called müllerianosis [13]. Other theories for the genesis of endometriosis include different mechanisms such as hematogenous metastasis, genetic predisposition or altered cellular immunity [1,2]. Nevertheless, all these theories remain speculative and no definitive evidences have been produced to demonstrate them. We speculated that, if the basis of endometriosis is an alteration during organogenesis, it would be possible to see ectopic endometrial tissue mislocated outside the uterine cavity of human female foetuses, possibly with a similar frequency found for endometriosis in the general population. Therefore, we decided to investigate the anatomy of the pelvic organs of a group of human female foetuses, collected at autopsy.

## Methods

We collected at autopsy 36 human female fetuses at different gestational ages, that did not displayed any visible alteration of the pelvic organs. The characteristics of the fetuses are depicted in Table 1. Pelvic organs were collected en-block, fixed in paraphormaldeyde and included in paraffin. We performed histological analysis of the pelvic organs for each fetus, using Hematoxylin/Eosin and Hematoxylin/Van Gieson staining. For immunohistochemistry 5–7 μm specimen sections embedded in paraffin, were cut, mounted on glass and dried overnight at 37°C. All sections were then deparaffinized in xylene, rehydrated through a graded alcohol series and washed in phosphate-buffered saline (PBS). PBS was used for all subsequent washes and for antiserum dilution. Tissue sections were quenched sequentially in 3% hydrogen peroxide in aqueous solution and blocked with PBS-6% non-fat dry milk (Biorad, Hercules, CA, U.S.A.) for 1 h at room temperature. Slides were then incubated at 4°C overnight at 1:100 dilution with the following antibodies: the affinity-purified rabbit antibody ERα for the oestrogen receptor (Santa Cruz, Santa Cruz, CA, USA; cat. # sc-542) and the mouse monoclonal antibody M11 for CA125(Dako Laboratories, Carpinteria, CA, USA). After three washes in PBS to remove the excess of antiserum, the slides were incubated with diluted goat anti-rabbit or anti-mouse biotinylated antibodies (Vector Laboratories, Burlingame, CA, U.S.A.) at 1:200 dilution in PBS-3% non-fat dry milk (Biorad) for 1 h. All the slides were then processed by the ABC method (Vector Laboratories) for 30 min at room temperature. Diaminobenzidine (Vector Laboratories) was used as the final chromogen and haematoxylin was used as the nuclear counterstaining. Negative controls for each tissue section were prepared by leaving out the primary antiserum. Positive controls constituted of tumour tissues expressing either the oestrogen receptor or CA125, were run at the same time. All samples were processed under the same conditions.

**Table 1:** Characteristics of the foetuses enrolled in this study

N°	Gestational age	Cause of death	Presence of ectopic endometrium
1	18 weeks	Voluntary abortion	Yes
2	24 weeks	Placental pathology	Yes
3	25 weeks	Placental pathology	Yes
4	16 weeks	Voluntary abortion	Yes
5	23 weeks	Placental pathology	No
6	15 weeks	Voluntary abortion	No
7	20 weeks	Voluntary abortion	No
8	newborn	Primary atypical pneumonia	No
9	newborn	Acute interstitial pneumonitis	No
10	16 weeks	Voluntary abortion	No
11	23 weeks	Placental pathology	No
12	14 weeks	Placental pathology	No
13	21 weeks	Voluntary abortion	No
14	20 weeks	Voluntary abortion	No
15	20 weeks	Voluntary abortion	No
16	18 weeks	Voluntary abortion	No
17	19 weeks	Voluntary abortion	No
18	16 weeks	Voluntary abortion	No
19	23 weeks	Placental pathology	No
20	25 weeks	Placental pathology	No
21	newborn	Acute interstitial pneumonitis	No
22	newborn	Primary atypical pneumonia	No
23	20 weeks	Voluntary abortion	No
24	19 weeks	Voluntary abortion	No
25	newborn	Cardiac malformation	No
26	newborn	Cardiac malformation	No
27	20 weeks	Voluntary abortion	No
28	23 weeks	Placental pathology	No
29	19 weeks	Voluntary abortion	No
30	newborn	Cardiac malformation	No
31	newborn	Cardiac malformation	No
32	19 weeks	Voluntary abortion	No
33	newborn	Acute interstitial pneumonitis	No
34	20 weeks	Voluntary abortion	No
35	newborn	Cardiac malformation	No
36	21 weeks	Placental pathology	No

Experiments were performed in compliance with the Helsinki Declaration and the protocols were approved by the ethics committee of the Fondazione Italiana Endometriosi.

## Results

**Figure 1:**
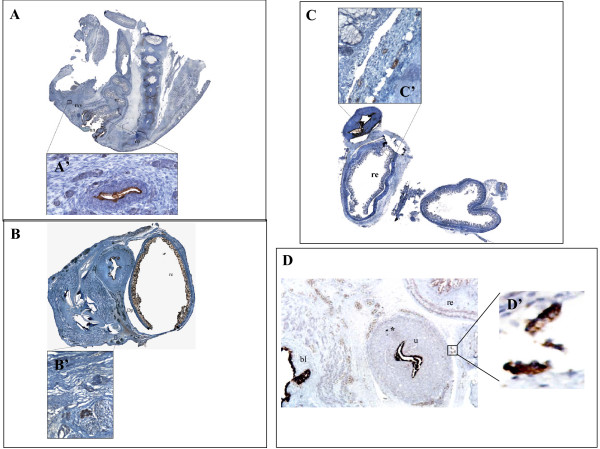
**Histological and immunohistochemical appearance of ectopic endometrium in four female human foetuses**. Panel A: A 25 weeks foetus showing an endometrial structure in the recto-vaginal septum; in the inset named A', the immunohistochemical expression of CA-125 of this structure at higher magnification is depicted. Panel B: A 24 weeks foetus showing an endometrial structure in the proximity of the Douglas poutch; in the inset named B', the immunohistochemical expression of oestrogen receptor of this structure at higher magnification is depicted. Panel C: A 18 weeks foetus showing an endometrial structure in the rectal tube at the level of muscularis propria; in the inset named C', the immunohistochemical expression of CA-125 of this structure at higher magnification is depicted. Note that the epithelium of the rectum is negative for CA-125. Panel D: A 16 weeks foetus showing an endometrial structure in the mesenchimal tissue close to the posterior wall of the uterus; in the inset named D', the immunohistochemical expression of CA-125 of this structure at higher magnification is depicted. Note that in the wall of the primitive miometrium is present a little group of endometrial cells positive for CA-125 (indicated by an asterisk), that could represent a primitive nest of adenomyosis. Abbreviations used: an (anus); co (coccyx); dp (Douglas' pouch); re (rectum); rvs (recto-vaginal septum); sc (spinal column); ut (uterus); bl (bladder).

In order to analyze the pelvic organs in their entirety, four sections were taken every 150 microns and stained for histology and for immunohistochemistry, as described in the method section. We have chosen, for immunohistochemisitry, CA125 and the oestrogen receptor, two well defined marker of epithelium of the female reproductive tract [1,14]. None of the selected cases displayed macroscopical or microscopical defects of the genital system. Indeed, we found in four foetuses (11% of cases), the presence of organoid structures outside the uterine cavity, clearly resembling the structure of the primitive endometrium and expressing both CA125 and oestrogen receptor. These structures were mislocated outside the uterine cavity and could not be ascribed to any normal anatomical formation. In particular, the locations of these endometrial structures were: in the recto-vaginal septum, in the proximity of the Douglas pouch, in the mesenchimal tissue close to the posterior wall of the uterus, in the rectal tube at the level of muscularis propria, and in the wall of the uterus. To note, these anatomical sites are common location for endometriosis in women [15]. The exact anatomical distributions and the histological appearances of these epithelial structures are depicted in detail in figure 1. We conclude that these structures must be ascribed to differentiated endometrial tissue, misplaced outside the uterine cavity during the earlier steps of organogenesis. It is possible to suppose that this ectopic endometrium would remain quiescent and, therefore, undetectable until puberty, when different stimuli, and among them the hormonal inputs, would cause its re-growth (as it is the case for the eutopic endometrium) and, consequently, the onset of the symptoms of endometriosis.

## Discussion

Despite the fact that Sampson's theory of retrograde menstruation/transplantation is still the most popular and accepted pathogenetic mechanism of endometriosis, several clinical and experimental evidence seems to contrast this hypothesis. There is, for example, no evidence *in vivo *or *in vitro *that endometrial cells present in the peritoneal fluid during menstruation can attach to and invade the peritoneal surface [16]. Furthermore, it has been shown that endometrial cells are not commonly present in peritoneal fluid [16-18]. Additionally, the fact that 90% of women have retrograde flow but less than 15% of women develop endometriosis and the presence of the disease in early puberty, further contrast the validity of the theory [18]. Finally, this theory fails to explain the presence of endometriosis in such remote areas as the lungs, skin, lymph nodes, breasts [1,2]. Interestingly enough, there are some studies showing higher prevalence of endometriosis in patients with Müllerian anomalies [19]; moreover, the existence of choristoma composed of müllerian rests, named müllerianosis, has been postulated [13]. In recent years, several evidence suggested that exposure to environmental toxicants possessing estrogenic activity, the so-called endocrine disruptors, resulted in endometriosis [20]. Although the epidemiological evidences are not conclusive to date, animal and experimental investigations have provided a basis for the proposed association between estrogenic contaminants exposure and endometriosis [21]. Nevertheless, the mechanism(s) underlying this potential association are poorly understood. The proper function of the normal human endometrium relies on well organized cell-cell interactions regulated locally by cytokines and growth factors under the direction of steroid hormones. The onset and progression of the disease processes of endometriosis may result from disruptions of this well balanced cellular equilibrium, that would cause the interruption of some organizational events associated with development of the neonatal uterine wall [21]. To the best of our knowledge, this observation is the first direct evidence in human female foetuses of the presence of ectopic endometrium outside the uterine cavity. Our data sustain the müllerianosis hypothesis of an embryological origin for endometriosis, suggesting alterations in the fine tuning of female genital structures organogenesis, possibly caused by environmental toxicants. Interestingly, the percentage of foetuses analyzed in our study, that displayed the presence of ectopic endometrium is very similar to the prevalence of women suffering for this disease in the general population [1-3]. This further suggests a strict link between embryological abnormalities and onset of the disease, even if the number of foetuses analyzed is too small in order to reach definitive conclusions. Further studies are urgently required in order to better define the molecular mechanisms underlying this phenomenon. In particular, *ad hoc in vitro *and *in vivo *models should be set up to analyze the effects on cell homeostasis and on the morphogenesis of the female genital system of different endocrine disruptors. Considering that, based on epidemiological studies, women with endometriosis have an increased risk of different types of malignancies, especially ovarian cancer and non-Hodgkin's lymphoma [1], the implications of these findings could be very important also in the oncology field.

## Conclusion

The clinical and therapeutic implications of this observation are straightforward. Endometriosis could not be regarded as a recurrent disease, therefore surgery, if complete can be considered curative and it would be not justified post-operative hormonal treatments. Nevertheless, it must be underlined the fact that other pathogenetic mechanisms for the genesis of endometriosis can not be completely ruled out by these observation, even if, to date, there are no direct evidence of their validity.

## Competing interests

The authors declare that they have no competing interests.

## Authors' contributions

PGS and AB conducted the work, analyzed the data and wrote together the manuscript. FB performed the histological and immunohistochemical analysis. RB, FB and MDA performed the autopsies. MDF performed the immunohistochemical staining.
